# Is explaining more like showing or more like building?—Agency in metaphors of explaining

**DOI:** 10.3389/fpsyg.2025.1628706

**Published:** 2025-10-15

**Authors:** Philip Porwol, Ingrid Scharlau

**Affiliations:** Department of Psychology, Faculty of Arts and Humanities, Paderborn University, Paderborn, Germany

**Keywords:** explaining, conceptual metaphor theory, metaphor, co-construction, agency

## Abstract

Explanations play a crucial role in knowledge transfer and meaning-making and are often described as a co-constructive process in which multiple agents collaboratively shape understanding. However, the metaphors used to conceptualize explaining may influence how this process is framed. This study investigates the extent to which the co-constructive nature of explaining is represented in explaining metaphors. Using a systematic analysis of agency, we examined how these metaphors depict the explanation process and the roles of the agents involved. We established that explaining metaphors lack collaboration between explainer and addressee, constructiveness of the process, as well as bidirectionality and iterativeness. In light of current research on metaphorical framing, the study thus highlights the risk that such explaining metaphors may reinforce a non-co-constructive perspective on explaining and a top-down approach in the development of AI systems as well as other areas.

## 1 Introduction

Explanations are pervasive in our everyday lives. On account of their ubiquity, explaining processes are a significant research topic, even more so after the technological progress of machine-learned artificial intelligence (AI; Rohlfing et al., 2021). The development of machine-learned AI systems is rapid. AI is increasingly used in different societal contexts, such as education (Zhai et al., 2021), medicine (Liu et al., 2021), law (Lai et al., 2024), or finance (Cao, 2022). Machine-learned AIs are opaque to laypeople as well as experts. Their increasing use in high-stakes domains requires them to be explainable. Thus, the call for *explainable AI* (XAI), which aims to make AI systems accessible, understandable, and criticizable for humans, is requested by many.

Researchers have provided different advanced theoretical frameworks to make AI more accessible (Miller, 2019). For instance, Ehsan and Riedl (2020) argue that XAI should be more human-centered (HCXAI). In contrast to other theoretical frameworks, HCXAI places significant emphasis on the interaction between AI and the social and contextual factors that influence it. Similarly, Sokol and Flach (2020) posit that more interactive explanations have the potential to increase the transparency of AI systems. Further, they argue that one-directional explanations often fail to meet the diverse needs of users and suggest that interactive explanations can address this issue.

Based on these observations, Rohlfing et al. (2021) point out several limitations in recent research on XAI and explanation and propose the theoretical framework of co-constructivity that addresses these shortcomings. In short, co-constructivity is characterized by a bidirectional and iterative explanation process in which the addressee is actively constructing the explanation in collaboration with the explainer. The agents negotiate both the goal and what is needed to know during the explanation process. In general, the co-constructive framework is characterized by a high agency of both agents involved. Following Rohlfing et al. (2021, 2025), we argue that a more co-constructive approach in human-computer interaction would improve the understanding of AI systems and the quality of the explanation processes. Interestingly, though, most existing approaches to XAI consist of unilateral statements by experts or systems in which the addressees have at most options of personalization, but neither the possibility of actively participating in the explanation nor in identifying the explanandum (Miller, 2019). The tendency of system developers and researchers to develop explanations one-sidedly from the perspective of the explainer seems to be pervasive, and interactive, social, or co-constructive approaches are only slowly and incompletely gaining acceptance.

In the present paper we ask which assumptions about explaining can be found in cultural thinking. We critically scrutinized core assumptions about the explaining process through metaphors in language. It is possible to draw conclusions about cognition through language because metaphors reflect “powerful cultural interpretation patterns” (Schmitt, 2024, p. 220). This consideration is based on the idea that language, culture, and mind are interdependent (Sinha, 2017). This means that we can – to a certain extent – understand conceptualizations of explaining through the examination of figurative language about explaining. Considering the significance of explanations and the use of AI in educational, medical, and judicial contexts (among others), we argue that it is crucial to ensure that the language about explanations is apt. Language patterns, such as metaphors, can contribute to misunderstandings of scientific content (Harrison and Treagust, 2006; Taylor and Drewsbury, 2018) as well as activities and practices (e.g., Hager and Hodkinson, 2009; Paulson and Armstrong, 2011). Explanations or explaining might be one example.

Because metaphors serve as potent representations of cultural and cognitive patterns, they offer a unique perspective into how we conceptualize explanation processes. Metaphors also make it possible to identify the limitations of language and use them as a basis for technological progress. In the present study we will answer the following questions: Which metaphors are commonly used to conceptualize the explaining process? How are the explanation process and the corresponding agents depicted through metaphor? With agency being a central component of co-constructivity, we more specifically asked how the agency of the agents is conceptualized. Overall, we thus study how common explaining metaphors support or impede the co-constructive approach to explaining.

For this purpose, we conducted an empirical study in which the participants produced metaphors for the process of explanation. These metaphors were analyzed with a structured method to elaborate the roles and the agency implied in the metaphors. In the following, we first introduce the notion of metaphor in the context of cognitive linguistics. We then differentiate the necessary schematic structures of metaphor and present studies on cognitive mechanisms and effects of metaphor to show how and to what extent metaphors can influence people’s thoughts. Lastly, we explain the method we used for examining agency, namely transitivity analysis.

### 1.1 Conceptual Metaphor Theory

In the field of cognitive linguistics, Lakoff and Johnson (1980, 2003) established *Conceptual Metaphor Theory (CMT).* It continues to be the most significant influence on scientific discourse on metaphor (for a bibliometric study, see Yuan and Sun, 2023), although after the establishment of *CMT*, several other metaphor theories have been developed in distinction to or as an extension of Lakoff and Johnson’s theory (for more information on the metaphor theories, see Kövecses, 2023; Steen, 2007, 2008). Numerous scholars have argued that metaphors are ubiquitous in everyday language and technical language (Gibbs, 2008; Lakoff, 1993; Lakoff and Johnson, 1980, 2003; Steen, 2011). Building on this theoretical work, an empirical study by Steen (2011) points out that 13.6% of the words in everyday discourse are metaphorical.

Lakoff and Johnson (1980, p. 5) define metaphor as “understanding and experiencing one kind of thing in terms of another.” Metaphors go beyond the level of linguistic expression; they are “systems of concepts in form of mappings between conceptual gestalts” (Kövecses, 2022, p. 34). Therefore, metaphors are ubiquitous not only in language but also in thought – the conceptual system in which individuals think and act is metaphorical as well (Lakoff, 1993; Lakoff and Johnson, 1980, 2003). According to *CMT*, the utilization of metaphors is not arbitrary, but rather indicative of shared cognitive structures. These structures originate in conceptual metaphors, which themselves emerge from recurring bodily experiences in the early stages of life (Lakoff and Narayanan, 2025).

Metaphors consist of a target domain, a source domain and mappings. The source domain, a concrete concept, “lends” properties to the target domain, which is a more abstract concept (Kövecses, 2021; Lakoff, 1993). The mappings between the conceptual target and the source domain can be defined as “systematic conceptual correspondences” (Kövecses, 2021, p. 193). In order to explain this in more detail, let us examine this sentence about explanations: “Humans are driven to acquire and provide explanations” (Keil, 2006, p. 227). In this sentence, an explanation is linguistically realized as an object that is given or acquired. In the corresponding metaphorical concept, explaining is giving,^1^ the abstract target domain, explaining, is metaphorically realized through the more concrete source domain, giving. In the source domain of giving, there is an agent who hands over a pre-existing object to a recipient. The object changes ownership from the possessing agent to the receiving agent. This structural information is mapped onto the target domain explaining: The explainer owns an object and gives this object to the addressee, who is then the new (or an additional) owner of the object. Because of the metaphorical correspondences, explaining is understood as the act of passing something pre-existing on to someone else.

Kövecses (2021, 2022, 2023) expands the foundational ideas of CMT and provides a framework of stability and variability of metaphors across cultures. While many metaphorical expressions vary culturally, many, but not all, conceptual metaphors seem to be almost universal across cultures (Kövecses, 2010). Examples of the latter are knowing is seeing or more is up. Kövecses argues that such metaphors are grounded in universal embodied experience. The more variable metaphors are dynamically shaped by contextual factors. Kövecses (2021) proposes the situational, bodily, the discourse and the conceptual-cognitive context as important factors.

Conceptual metaphors can be defined at different levels of schematicity. In order to understand the general ways in which the target explaining is conceptualized by metaphors we need an appropriate level that is neither too schematic nor too specific. Kövecses (2017, 2021, 2022, 2023) offers a comprehensive framework to distinguish conceptual metaphors by their schematicity. In the following, we argue that the level of domains and especially the level of frames are adequate for our purposes.

Domains are propositional conceptual structures (Kövecses, 2017). Langacker (1987, p. 488) defines domains as “a coherent area of conceptualization relative to which semantic units may be categorized.” For instance, conceptual metaphors like communication is transfer, ideas are objects, complex abstract systems are buildings, or ideas are perceptions derive from domains (Kövecses, 2017). According to Sullivan (2013, 2017), domains are composed of multiple frames and subframes.

Ruppenhofer et al. (2010, p. 5) define a frame as “a script-like conceptual structure that describes a particular type of situation, object, or event along with its participants and props.” Frames contain specific information about roles and the relationship between roles, fleshing out the corresponding domains (Kövecses, 2017). They are also the foundation for grammatical constructions (Fillmore, 1982). In the frame giving, for instance, there is the giver, the recipient and the object that is transferred. Other examples of conceptual metaphors that are founded on frames are knowing is seeing or understanding is grasping (Kövecses, 2017). It is worth noting that the distinction between domains and frames is not evident in all cases because there are multiple views on the differentiation between domains and frames (Cienki, 2010; Sullivan, 2013, 2017). Dancygier and Sweetser (2014) as well as Kövecses (2017) argue that the degree of schematicity enables a distinction between frames and domains, as domains are more schematic and less specific than frames – a view that we adopt in our analysis.

Note that conceptual metaphors of all levels have to be distinguished from metaphorical expressions that are metaphorical words, phrases or sentences, that is, “surface realizations” of the conceptual metaphors (Lakoff, 1993, p. 203). Metaphorical expressions allow conceptual structures to be formed and analyzed (Schmitt, 2024).

In the present research, we reconstructed both frames and domains. This differentiation is relevant for further analysis because it determines the methodology used in this research (Kövecses, 2017). In our metaphor analysis, the conceptual structure of frames allowed us to determine the agency of the specific metaphoric actions and domains, which were established, to group frames and to expand the interpretative framework. We also analyzed metaphorical expressions because, as argued by Karsten et al. (2022), the exact linguistic expression may change the meaning of a metaphor. This is the case, for instance, when a verb such as *giving* or *moving* is used in the passive voice. This reduces the agency implied in the metaphor.

### 1.2 Metaphorical framing

As described earlier, Lakoff and Johnson (1980, 2003) claim that metaphors are elements of shared and coherent thought structures. In addition to this claim, they also identified the mechanisms of highlighting and hiding. Metaphors can emphasize certain aspects of the target concept (highlighting) and obscure other aspects and make them more difficult to perceive (hiding). For example, the metaphorical conceptualization explaining is giving emphasizes the interpersonal aspect of explaining while obscuring the active role of the addressee who in this conceptual structure is a passive receiver of the object.

Highlighting and hiding mechanisms allow metaphors to influence thoughts and attitudes. A significant amount of research has been conducted on cognitive metaphor framing effects on these processes and states (e.g., Flusberg et al., 2024; Thibodeau et al., 2017, 2019). Empirical research has shown that metaphors can influence people’s thinking about topics such as immigration (Chkhaidze et al., 2021), artificial intelligence (Khadpe et al., 2020), teaching (Wong et al., 2022), climate change (Flusberg et al., 2017), or cancer (Hendricks et al., 2018). The most prominent definition of framing originates from Entman (1993, p. 52):

“Framing essentially involves selection and salience. To frame is to select some aspects of a perceived reality and make them more salient in a communicating text, in such a way as to promote a particular problem definition, causal interpretation, moral evaluation, and/or treatment recommendation for the item described.”

Above, we have already mentioned a possible framing effect: If somebody talks of “*giving* an explanation” (for instance a future XAI system that is about to explain its recommendation for a medical intervention), it may make a difference if it starts with “Let me *give* you an explanation of the main reasons for this recommendation” or with “Let me *guide* you *through* an explanation of the main reasons for this recommendation.”

In addition to metaphorical framing, which is the framing through the usage of metaphors – giving and walking in the example –, there are other framing effects which can reinforce or mitigate effects of metaphorical framing (for a detailed overview, see Flusberg et al., 2024). One of them is grammatical framing. Grammatical framing involves the manipulation of sentence structure, tense, aspect etc. These linguistic aspects may also frame the content of the sentence (Flusberg et al., 2024). For instance, Fausey and Boroditsky (2010) reported that an agentive framing (“she flopped the napkin”) results in more blame and punishment than a non-agentive framing (“the napkin flopped”). Similarly, eyewitness memory seems to be influenced by the grammatical system of one’s language (Fausey and Boroditsky, 2011; Fausey et al., 2010). These results highlight the relevance of investigating the linguistic realizations of metaphors. This may be especially important regarding explaining metaphors where – in the co-constructive framework – both the explainer and the addressee are assumed to be active agents of the process.

### 1.3 Agency analysis

Based on the results of both agentive and metaphorical framing, we argue that it is important to analyze the language of explaining, more specifically, common metaphors of explaining and their usage. On the one hand, these aspects of language of explaining may reveal cultural thinking about explaining that is too self-evident or belongs too much to the respective culture to be a target of reflection. On the other hand, common metaphors may influence how explanations are perceived and designed in everyday and professional communication as well as in technical systems.

An important aspect of co-constructive explaining is, as mentioned above, the high agency the agents. According to Helfferich (2012), agency encompasses the capacity to act, the attribution of power and the influence of agents upon their environment. To investigate the agency in conceptual structures and their corresponding linguistic realizations, which can have framing effects on the explanation process, we used transitivity analysis (Hopper and Thompson, 1980; Karsten et al., 2022). Transitivity analysis is a structured lexico-grammatical method for investigating the conceptual structures, their corresponding linguistic realizations and the implications of metaphors. It is especially useful when actions and their associated agency are analyzed (e.g., Scharlau et al., 2019, 2021). The metaphorical content is examined based on a linguistic theory with the help of semantic and syntactic parameters.

Hopper and Thompson (1980, p. 251) describe transitivity as “a global property of a whole clause such that an activity is “transferred” from an agent to a patient.” Transitivity can therefore be seen as a linguistic concept of agency. In addition, Charteris-Black (2018) argues that transitivity provides information about the relationship between an agent and an entity as well as the ongoing action and can also indicate how the agents and their agency are highlighted or hidden. Therefore, we argue that the agency of actions can be determined by means of transitivity analysis (Karsten et al., 2022).

Hopper and Thompson (1980) specify ten semantic and syntactic parameters that can be used to determine the agency of an action. The parameters have two poles which are related to high or low transitivity. Table 1 briefly presents the transitivity categories and their poles.

**Table 1:** Transitivity parameters by Hopper and Thompson (1980).

Parameters	Description
Participants *Several participants vs. one participant*	Activities that involve both a subject and an object are considered transitive. Activities that only involve a single participant are considered intransitive.
Kinesis *Action vs. state*	In contrast to states, actions are categorized as transitive, as one can exert influence on an object through actions.
Aspect *Telic vs. atelic*	Actions that pursue a clear goal are categorized as transitive, while those that do not have a clear goal are classified as intransitive.
Punctuality *Punctual vs. non-punctual*	Punctual actions without a clear transition phase between the beginning and the end are considered transitive.
Volitionality *Volitional vs. non-volitional*	Purposeful activities are categorized as transitive, in contrast to unconsciously performed actions.
Affirmation *Affirmative vs. negative*	An affirmative formulation is regarded as transitive and negative formulations are regarded as intransitive.
Mode *Realis vs. irrealis*	While expressions in the subjunctive are categorized as intransitive, expressions in the indicative are considered transitive.
Agency *High vs. low*	Animate subjects have a higher agency and are therefore ascribed a higher transitivity than inanimate agents.
Affectedness of the object *Totally affected vs. not affected*	If an object of action is modified by an action, the activity is considered transitive; if an object of action is hardly or not at all hardly affected, the action is categorized as intransitive.
Individuation of the object *Highly individuated vs. not individuated*	The transitivity of an activity is high if there is a concrete and individuated object that can be influenced. Abstract objects can be influenced to a lesser extent so that actions that affect abstract objects are more intransitive.

As mentioned above, we want to examine whether the agency of common explaining metaphors corresponds with the agency of the co-constructive framework of explaining. For our analysis we focus on the parameters *participants, punctuality,* and *affectedness of the object* because each of the parameters shows an essential aspect of the co-constructive explanation process. In order to appropriately describe and compare the metaphorical actions to the co-constructive notion, we had to slightly adapt the parameters of Hopper and Thompson.

Hopper and Thompson’s *participants* examines the number of participants, both animate and inanimate, involved in a clause but it does not provide information about the number of human participants and the extent of activeness of the agents, which are both essential within our target domain. Therefore, we extended the parameter *participants*: First, we analyzed the number of human agents in the clause. Second, we examined whether both human agents engage actively in the explaining process. Both the number of agents and the activeness of the agents is essential because the co-constructive explanation process is seen as a collaborative process between two active participants.

For Hopper and Thompson (1980), a punctual action is more transitive than a non-punctual one. While agreeing with their notion in general, we still made two changes that were essential for the target domain we analyzed here. Firstly, we analyzed whether the action involves bidirectionality because both explainer and addressee construct the explanation. Secondly, we included iterativeness in our analysis. We decided to make these changes for the analysis because in the theoretical framework of co-constructivity, the explanation process is iterative and bidirectional. Based on this, we regard a longer-lasting collaborative action to be more agentive than a punctual one. To emphasize this difference between our understanding of agency in the target domain of explaining and the original concept of Hopper and Thompson, we call this parameter *temporality*.

The constructiveness of the explanation process should be reflected in the parameter *affectedness of the object*. This parameter represents object changes caused by the action of the agents. If an object itself is altered, transitivity is high and if the object is not modified due to the action, the action is considered intransitive. For example, if the object is moved from one place to another, the affectedness is low.

In the analysis presented below, the agency of explaining metaphors is compared to the agency of the co-constructive framework with the help of these transitivity parameters.

## 2 Materials and methods

300 German metaphor texts and 263 English metaphor texts were collected online in 2022 and 2023 via the service provider Prolific. The participants were at least 18 years old and were native speakers of German or English, respectively.

In accordance with the method *Elicited Metaphor Analysis* (Low, 2015), the participants were explicitly asked to produce a metaphorical expression about explaining in response to the following prompt:

1. Imagine you meet a peer who, for some reason, has no understanding of what “explaining” means.
2. Please choose an image/analogy/metaphor for “explaining” and use it to explain to your peer what “explaining is like.
3. Write your explanation in the box below. Start your text with the sentence “Explaining is like….”
4. What about your image/analogy/metaphor fits your concept of “explaining” and what does not? There is no right or wrong when answering these questions. We are simply interested in what you imagine “explaining” to be like in as much vividness as possible.

In order to create a basis for further metaphor interpretation, a standardized method of metaphor identification of the elicited metaphor texts had to be applied. We decided to use the metaphor identification method developed by Steen et al. (2010) called *MIPVU*. In this method, the meaning of every single lexical unit is compared to the basic meaning found in dictionaries. As recommended by Steen et al. (2010), we used dictionaries for identifying the basic meaning. For English texts, this was the *Macmillan English Dictionary for Advanced Learners* (Rundell, 2007). The *Oxford Dictionary* (Oxford University Press, n.d.) was used as a supplement. Comparable dictionaries of the German language were used; the *Digitales Wörterbuch der Deutschen Sprache* (Berlin-Brandenburgische Akademie der Wissenschaften, n.d.) and as an addition the *Duden* (Dudenredaktion, n.d.).

Among the metaphors, we analyzed only those related to the target domain explaining. Since our focus was on agency of the explaining process and the usage of transitivity analysis, only verbs and nominalized verbs were identified and coded. For each word, we compared whether the basic meaning corresponded to the meaning of the units in the metaphor texts. If this was not the case, the word was identified as a metaphor.

The following example illustrates the subsequent process of analysis. Based on the basic meanings of the *Macmillan* (Rundell, 2007), the verbs in italics were identified as metaphorical.

- “A good explainer *adjusts* their approach.”
- “Such as analogies [.] that an explainer might use to help *shape* the information into a clear and understandable form.”
- “A good explainer will *tailor* their approach to the person.”

Frames were first reconstructed by grouping the similar meanings of the lexicons used. Because the Macmillan definitions of the terms *adjusting* (“To change something slightly in order to make it better, more accurate, or more effective”), *shaping* (“To form something into a particular shape”) and *tailoring* (“To make or change something especially for a particular person or purpose”) are quite similar, they were summarized as one frame that we called adjusting. Frames were included in the analysis if at least 3 metaphors allocated to them were identified in the 300/269 texts in our corpus.

Frames were then grouped into domains. The grouping of the domains was conducted using the Master Metaphor List (Lakoff et al., 1991), a collection of basic metaphorical concepts, as a reference. In addition to the frame adjusting, several other frames that focus on the modification of objects were identified, such as removing or putting together. These were reconstructed as the domain modification. Again, we decided to include only frames that were present in at least 3 of the texts. Domains were only derived if there were at least two frames which were assigned to the domain.

The frames were added to a coding manual which contains the domains, frames, examples of the metaphorical concepts, and definitions of the metaphorical expressions. With the help of the coding manual, we conducted further reviews of the texts to identify more metaphorical expressions, until no further frames and domains could be reconstructed, and no more metaphorical expressions could be found.

The software MAXQDA (for more information on the usage for qualitative research, see Kuckartz and Rädiker, 2019) was used for the coding of the metaphorical expressions. In each target domain, two researchers coded the metaphors separately. If a metaphor concept occurred several times in a metaphor text, only the first time was coded because we were interested in the frequency of metaphorical concepts across the dataset rather than the frequency of concepts within a text. The intercoder agreement was determined with the help of MAXQDA by calculating the code overlaps in the text.

Once all metaphorical expressions had been identified and the frames reconstructed and coded, they were analyzed on a conceptual and linguistic basis using the transitivity parameters mentioned earlier. The meanings given in the dictionaries were used as the basis for our analysis. The agency of the parameters was compared to the agency of the co-constructive approach. If the agency did not match the co-constructive approach, the parameter was assessed as negative. We provide an open-access corpus with annotated verb metaphors at the Open Science Foundation.^2^ The coding manuals with the domains and frames of explaining are made available in the Supplementary materials.

## 3 Results

In the following, we first present the metaphorical domains identified in the data. Secondly, we present the agency of the frames to answer the question of how agency is conceptualized and whether it fits a co-constructive understanding of explaining. Finally, we compare the English and the German metaphors. For the sake of brevity, we present the analysis of the English corpus in detail; the German results differ little.

For a full overview of the conceptual structures and the ratings of all parameters in English and German. The intercoder agreement is 86% for the German data and 91% for the English data, resulting in a Cohen’s *κ* of 0.85 in the German dataset and 0.9 in the English dataset. According to Landis and Koch (1977) this is rated as an almost perfect agreement. For the final analysis, all disagreements were remedied by one of the coders.

### 3.1 Analysis of the domains

**Figure 1:**
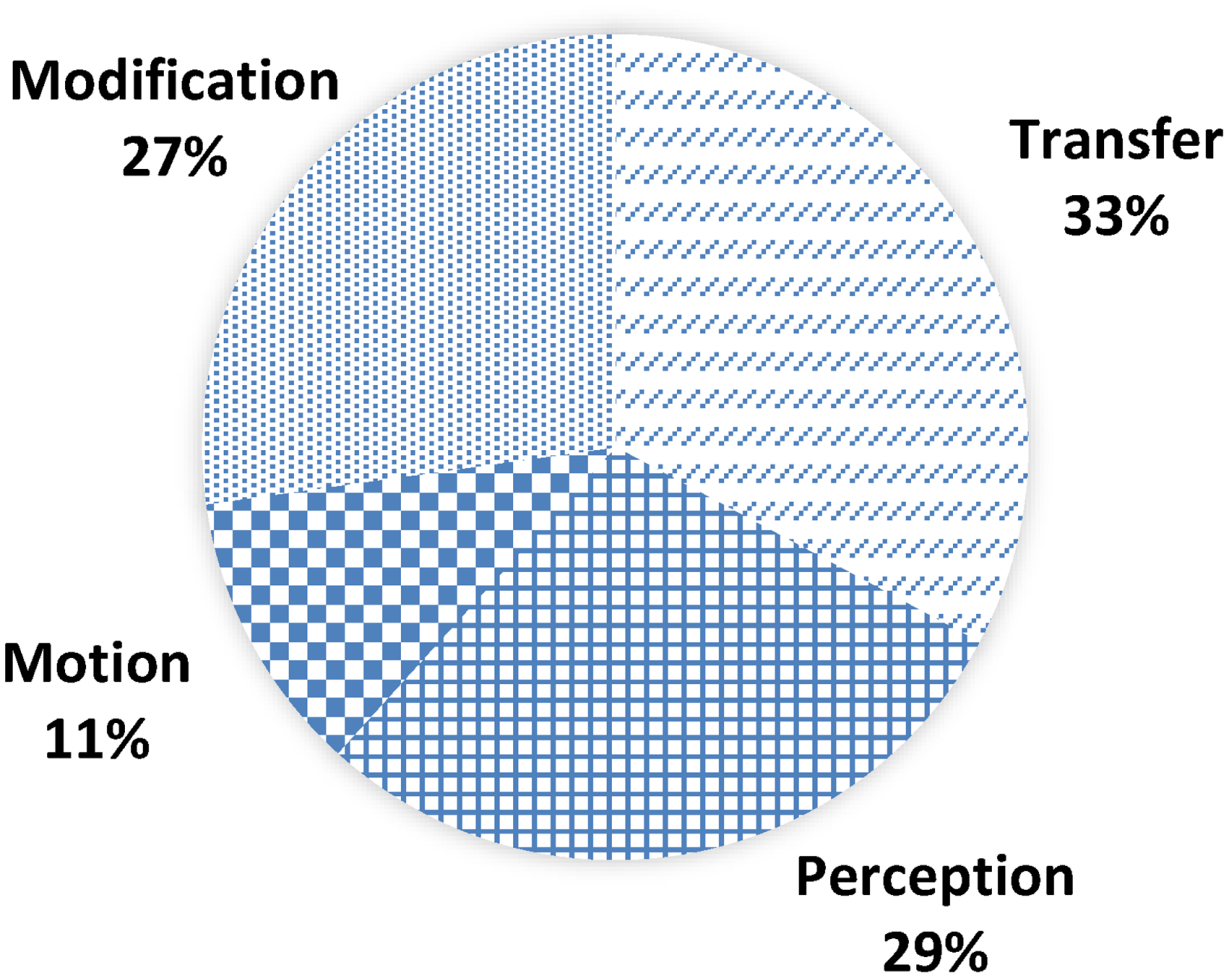
Distribution of conceptual domains of explaining across the data.

As Figure 1 illustrates, four domains were reconstructed in our data: transfer, modification, perception and motion. The percentages in Figure 1 and in the following tables are determined by the number of texts in which the domains or frames were present.

With 175 cases out of 263 in the English data, the domain transfer was most common. Three frames were allocated to this domain, namely giving, conveying, and delivering. transfer has a close connection to the conduit metaphor, which was prominently discussed by Reddy (1979). The premise of the conduit metaphor is that communication is conceptualized as sending information from one person to another with the help of a conduit. The explainer formulates his thoughts, packages them with linguistic expressions and transmits them via a conduit to an addressee who then unpacks the thoughts (Reddy, 1979; Lakoff and Johnson, 1980). Thus, the explaining process is realized as a transmission of objects between two agents. In general, knowledge, the explanation itself or understanding is given.

The domain perception was present in 154 out of 263 metaphor texts and includes the frames showing, clarifying, seeing and illuminating. The domain has a close connection to Lakoff and Johnson’s (1980, 2003) conceptual metaphor understanding is light/seeing or ideas are perceptions (Lakoff et al., 1991). In this spectrum of frames, explaining is realized as an action of making an object more visible. The visibility of the object was originally obscured by factors such as darkness or gloom. The explaining process in this domain is conceptualized as inducing a change of perspective of the addressee.

modification was the third most common domain with a frequency of 145 including 12 different frames. This domain summarizes actions that involve the alteration of an object or a structure: Explaining involves altering objects, creating new entities or an organization of systems. Exemplary frames are adding, connecting, removing or opening. This domain derives from the conceptual metaphors thinking is manipulating an object, ideas are objects and thinking is building/forming/shaping (Lakoff et al., 1991).

The domain motion only occurred 59 times and covers the frames guiding, walking and taking. Motion is primarily connected to the conceptual metaphors change is motion and action is motion, or more specifically, guided action is guided motion and (Lakoff et al., 1991). The explanation process is conceptualized as an act of moving toward or taking someone to a specific destination.

Some frames could not be assigned to the four domains mentioned above. These are the frames introducing, helping, checking and simplifying. Different to the other frames, these frames are lower in imagery and structural mapping. For instance, the frame helping involves the metaphors *helping* and *aiding*, which are defined in the Macmillan as “To give someone support or information so that they can do something more easily” and “To make it easier for someone to do something.” In relation to explaining, the frame helping is low in imagery and structural mapping.

Due to their low imagery and weak metaphorical mappings, and because they could not clearly be assigned to a conceptual domain, we decided to exclude these frames from the analysis. This does not imply that these metaphors are unimportant or would not cause framing effects; they would merely contribute little of a systematic nature to our analysis of agency.

### 3.2 Analysis of frames with transitivity analysis

The most common frames in the individual areas and their agency ratings are presented in the following. In the domain transfer, the most frequent conceptual structure, both in the English and in the German dataset, was giving with 140 occurrences (appearing in more than half of the texts). This metaphorical concept usually involves two human participants: One person hands an object to another person. In contrast to the co-constructive framework of explaining the recipient, however, is not active in the action. Giving is a punctual and unidirectional action and thus does not reflect the iterative character of a co-constructive explanation. Further, the object merely changes location and is not influenced in any other way. In a co-constructive explanation, however, both participants are actively modifying the explanation. Generally, the frames of the domain transfer lack *affectedness of the object* and conceptualize the actions as punctual or short and neither bidirectional nor iterative (Tables 2, 3).

**Table 2:** Evaluations of agency parameters across domains.

Domains	Participants	Temporality	Affectedness of the object
transfer	~	X	X
modification	X	~	✓
perception	X	X	X
motion	~	~	X

**Table 3:** Distribution of frames of the English data across domains with evaluations of the relevant agency parameters.

Domains	Frames	Frequency	Participants	Temporality	Affectedness of the object
transfer	Conveying	8%	~	X	X
	Delivering	5%	~	~	X
	Giving	53%	~	X	X
modification	Adding	4%	X	~	✓
	Adjusting	5%	X	~	~
	Breaking Down	16%	X	~	✓
	Building	4%	X	~	✓
	Connecting	2%	X	~	✓
	Creating	6%	X	~	✓
	Making	2%	X	~	✓
	Opening	3%	X	X	~
	Painting	6%	X	~	~
	Putting Together	5%	X	~	✓
	Removing	3%	X	X	✓
	Solving	2%	X	X	✓
	Turning on	1%	X	X	~
perception	Clarifying	14%	X	~	✓
	Illuminating	3%	X	X	X
	Seeing	2%	X	X	X
	Showing	32%	~	X	X
motion	guiding	7%	~	~	X
	Taking	3%	~	~	X
	Walking	13%	~	~	X

X = parameter absent; ~ = parameter partially or ambiguously present; ✓ = parameter present.

The most common frame of the domain modification was breaking down with a frequency of 41 (16% of the texts). It summarizes all metaphorical expressions that describe separating an object into smaller parts. In this frame, the object is highly *affected*, because the act of dividing an object highly modifies the structure of the object. The act of breaking something down usually only involves a single agent (*participant*) and an object. Further, the duration of the action is relatively short, but the action can be done multiple times by the single agent (*temporality*). Regarding *affectedness of the object* the frame shows a high agency, especially compared to other concepts. Here, too, there only is partial agreement with the co-constructive approach, as there is little or no collaboration, bidirectionality and extendedness of the action of breaking down. The frames of the domain modification generally have a high *affectedness of the object* and a low degree of *temporality*. Typically, a single human participant carries out the actions; multiple human participants are rare in this concept. Note that the frames would allow for participation of several actors in the action. This was, however, rarely realized on the linguistic level.

showing, which was the most frequent concept of the domain perception at a frequency of 83 (one third of the texts), includes metaphorical expressions such as *portraying, pointing out, revealing* or *presenting*. This frame involves an agent who directs the gaze of another agent in a certain direction. The action is highly punctual and unidirectional. The action does imply multiple participants, but the perceiver does not contribute to the action. The object of action is not modified in any way, because an entity is merely put into focus. The agency of the frame showing thus does not correspond with the essential elements of explaining in the co-constructive framework. There is a lack of collaboration, the object is not co-constructed by both agents and the duration is short. Further, the action does not involve bidirectionality. As a rule, only the visibility of the objects of these metaphorical actions is modified and the actions are relatively punctual.

In the domain motion, walking appears 33 times in our data and thus in 13% of the texts. It contains the action of moving along a path. In this frame, there is no object that can be affected by the action of walking. Further, multiple agents can be involved, yet additional agents are generally passive co-participants rather than active collaborators. However, the duration of the activity is ongoing for an extended period of time. Regarding the parameters *participants* and *affectedness of the object*, the agency of the frame does not match the agency of co-constructivity, because there is no genuine collaboration and the path or the goal of the action cannot be modified by the agent. In *temporality*, the frame does not fully match the agency of the theoretical framework either. The iteration and bidirectionality of the process between two agents is not represented here. In general, the associated actions last for a longer period of time and the objects are not affected through the actions.

So far, we have analyzed the agency of the metaphorical concepts. As mentioned above, we can also analyze the specific linguistic expressions used by the participants in our data collection. This realization may or may not match the agency of the concept. To give an example: With respect to the frame of giving, one might say that the explainer hands over an element of the explanation to an addressee or that an explanation is given by a teacher. In terms of our analysis scheme, the first formulation is more agentive than the second one because it mentions the second participant and uses the active voice.

We considered such formulation peculiarities as an additional check of our conclusions. There was one dominant pattern, namely that the agency was reduced by the specific phrasing. More specifically, the number of participants decreased. For example, in the utterance “giving your explanation” a gerund is used to realize the metaphor. In such gerund phrases, the explainer is not realized linguistically and thus hidden. This also becomes apparent in the utterance “explaining seeks to give a more detailed expression of the subject matter.” The explanation or the explaining process are utilized as both acting subject and object, obscuring both the explainer and the addressee. Passive constructions can similarly decrease the human participants to zero. In the utterance “an understanding is revealed” no human participants are realized at all. Although limited in number, formulations of this kind which change the agency of the metaphors recurred throughout the data. We do not wish to focus on them here; they support our conclusion of low agency of explaining metaphors. Whether they produce linguistic framing effects would be the subject of a separate study.

### 3.3 Comparison of frames in the German and English data

Both the German and English texts contain frames of the domains transfer, modification, perception and motion. The majority of frames resemble one another in both languages – 20 of the 23 frames of the English data can also be found in the German data. They could be directly translated from one language into another. These corresponding metaphors are listed in Table 4. The agency analysis of the German data is, as expected, very similar to the analysis of the English metaphors described above. For example, the similar frames opening and öffnen are both non-collaborative, non-iterative and unidirectional and the object merely changes its location.

**Table 4:** Distribution of similar frames in the German and English data.

Explaining	Frequency	Erklären	Frequency
giving	53%	geben	28%
conveying	8%	vermitteln	17%
delivering	5%	liefern	3%
building	4%	bauen	5%
creating	6%	schaffen	5%
adding	4%	hinzufügen	2%
connecting	2%	verknüpfen	3%
breaking down	16%	zerlegen	4%
removing	3%	entfernen	2%
putting together	5%	zusammensetzen	3%
painting	6%	malen	5%
opening	3%	öffnen	5%
solving	2%	lösen	1%
turning on	1%	anschalten	1%
clarifying	14%	klären	7%
illuminating	3%	beleuchten	5%
guiding	7%	führen	5%
walking	13%	gehen	2%
taking	3%	mitnehmen	1%
showing	32%	zeigen	20%

With respect to differences, we identified more frames in the German data. Most of the additional frames were from the domains transfer and modification and their interpretation is compatible with the results presented above. There also were three frames in the English data set without direct equivalent in the German data set, all of them interpretable within our domains. The different frames in English and German can be seen in Table 5. The results of the analysis of the German data and Table 6, which shows the additional frames in both English and German.

**Table 5:** Distribution of different frames in the German and English data.

Explaining	Frequency	Erklären	Frequency
adjusting	5%	übertragen	2%
seeing	2%	transferieren	2%
making	2%	entfalten	1%
		füllen	1%
		greifbar machen	3%
		ordnen	2%
		platzieren	1%
		verbreiten	1%
		Begleiten	1%
		Suchen	1%

**Table 6:** Distribution of frames of the German data across domains with evaluations of the agency parameters.

Domains	Frames	Frequency	Participants	Temporality	Affectedness of the object
Transfer	Geben	28%	~	X	X
	Liefern	3%	~	~	X
	Transferieren	2%	~	X	X
	Übertragen	2%	~	~	X
	Vermitteln	17%	~	X	X
Modification	Anschalten	1%	X	X	~
	Bauen	5%	X	~	+
	Entfalten	1%	X	X	~
	Entfernen	2%	X	X	+
	Füllen	1%	X	~	+
	Greifbar Machen	3%	X	~	+
	Klären	7%	~	~	~
	Hinzufügen	2%	X	~	+
	Lösen	1%	X	X	+
	Malen	5%	X	~	~
	Öffnen	5%	X	X	~
	Ordnen	2%	X	~	~
	Platzieren	1%	X	X	~
	Schaffen	5%	X	~	+
	Verbreiten	1%	X	~	~
	Verknüpfen	3%	X	~	+
	Zerlegen	4%	X	~	+
	Zusammensetzen	3%	X	~	+
Perception	Beleuchten	5%	X	X	~
	Suchen	1%	X	~	X
	Zeigen	20%	~	X	X
Motion	Begleiten	1%	~	~	X
	Führen	5%	~	~	~
	Gehen	2%	X	~	X
	Mitnehmen	1%	~	~	~

X = parameter absent; ~ = parameter partially or ambiguously present; ✓ = parameter present.

## 4 Discussion

In view of the rapid development of AI and its increasing presence in diverse societal contexts, the importance of XAI and the enhancement of human-computer interaction when explaining AI is steadily rising. We argue that the implementation of social and co-constructive aspects proposed by Rohlfing et al. (2021, 2025) in AI systems is one important component of achieving these improvements.

The goal of the present research was to analyze explaining metaphors with the help of the co-constructive aspects of explaining to find out how the explanation process is conceptualized and whether the co-constructive approach to explaining is existent in the metaphors that are used for explaining. For this purpose, we evaluated the agency of explaining metaphors identified in an English and German dataset collected from native speakers and compared it to the co-constructive view on explaining. Specifically, we identified metaphorical frames of different domains and analyzed them using transitivity analysis, a structured method to analyze the degree of agency or effectiveness of the action in a verbalized event. In the present version of transitivity analysis, the presence and activeness of both explainer and addressee, the duration, iterativeness and bidirectionality of the action as well as the affectedness of the object were examined. Our analysis suggests that common explaining metaphors tend to limit a co-constructive understanding of explaining – in the English and in the German dataset. Their implications hinder a co-constructive understanding.

In more detail: The second participant is either only implicit or does not take an active role in the action (parameter *participants*). In the theoretical framework of Rohlfing et al. (2021), the addressee is necessarily co-constructing the explanation through collaborative actions and takes on an important role in the explanation process. Although the application to the notion of explaining is new, we are not the first to point out this mismatch. Reddy (1979) also draws attention to the passiveness of the addressee in the conduit metaphor. Also, the psycholinguist Clark (1996) argues that language (which is heavily involved in explaining) itself should be seen as a joint effort.

The analysis of the explaining process further supports the interpretation that explaining metaphors impede co-constructive aspects (parameter *temporality*). Most metaphorical actions are short-lived, unidirectional and non-iterative rather than iterative and bidirectional as in the co-constructive framework.

In most metaphors, the object remains unchanged by the metaphorical actions (parameter *affectedness of the object*). The most prevalent change of the objects is a change of location, which is typically realized by the domain transfer or motion. Further modifications are the illumination of objects or changing the viewing direction of the agent to ensure visibility. All of these, however, leave the object itself relatively unaffected. This suggests that the explanandum is typically treated as a predefined, rigid entity that is simply handed over rather than actively shaped in collaboration with the addressee. Again, there is a parallel in earlier discussions: Geeraerts (1993) critiques the conduit metaphor in a similar way, arguing that meaning should be understood as constructed through interaction rather than as a transfer of a fixed object.

An exception is the domain modification with frames such as opening, adjusting or adding. Objects are created from scratch or with the help of parts, they are connected, opened, certain elements are removed, they are adjusted or broken down. To a larger or lesser degree, these metaphors allow for the construction or even co-construction which is one – but only one – of the essential elements of Rohlfing et al.’s (2021) conceptualization of explaining.

In the context of XAI, the findings may explain why many current systems result in a predominantly top-down approach where explanations are “built” by experts or AI and “given” without engaging the addressee. It may also explain why progress in more interactive approaches is so slow, even though these have long been repeatedly demanded. The metaphorical conceptualizations we identified in the present research may be inadvertently built into systems by developers by means of their metaphorical conceptualizations of explaining. This could be all the more true given that the metaphors we have identified are so commonplace that they are probably not even perceived as specific ideas in most cases and therefore cannot be challenged. Metaphors are in fact widely used in technology (e.g., Wilson, 2024) – think of the *desktop*, *surfing*, the *web*, *chatting*, and the like, and often discussed as helpful (e.g., Kim and Maher, 2020). Researchers have, however, repeatedly pointed out that, although often fostering understanding, metaphors may lead to misconceptualizations that impair the use of technology and have suggested more appropriate metaphors (e.g., Gentner and Nielsen, 1996). Most of this research has addressed metaphors for elements of the technology itself, especially concerning human-computer interfaces. We take this approach a step further to XAI and the practices of explaining.

In order to align with the societal need for understanding, criticizing and co-constructing AI, XAI frameworks need to incorporate more human-centered dynamic, interactive, co-constructive elements that allow users to ask questions, provide feedback, and iteratively refine their understanding. As argued by Ehsan and Riedl (2020), Sokol and Flach (2020) and Rohlfing et al. (2021), this would support users as active participants in the explanation process. This would not only be beneficial to the explanation process but is also ethically urgent because it would empower users to critically and pro-actively engage with AI systems rather than passively receiving their inputs.

Yet, despite this need, we expect that contemporary AI systems are deficient regarding aspects of co-construction. This assumption is supported by research conducted by Lenke and Schulte (2025). In a workshop setting, the theoretical framework of co-construction was introduced. The interaction between ChatGPT and the participants was tested in a pre-post-test design, and the monitoring and scaffolding prompts were then compared. Following the workshop, the participants showed an enhancement in co-constructive prompts. Lenke and Schulte (2025) further posit that the occurrence of co-constructive interaction is not attributable to ChatGPT itself, but that the responsibility for causing such interaction lies with the addressee.

The relevance of our study is not limited to the XAI context. We have focused on it because the considerations of Ehsan and Riedl (2020), Sokol and Flach (2020) and especially the framework of Rohlfing et al. (2021) provided a very precise idea of claims and XAI with which we were able to compare the metaphors. But, of course, some of this can be transferred to educational contexts. Prevailing explaining metaphors may reinforce teacher-centered practices. Educators might adopt methods that prioritize delivering content rather than fostering active dialogue. Duru (2015) for instance has demonstrated that most teacher metaphors of teacher-training students reflect teacher-centered beliefs.

With its critical focus, the present analysis does, in a very specific, empirical way, what computer scientist Agre – to mention only a single researcher – aimed at in his Critical Technical Practice (Agre, 1997). We analyzed metaphors for highlighted and neglected aspects in the notion of explaining. The first goal is to become aware of these metaphors, as a precondition for attempts to adjust or change them and then finally improving explaining practices in XAI and the quality of human-computer interaction. One might take this approach much further to what scholars in, above all, Science and Technology Studies and feminist studies have done (e.g., Latour, 1987; Haraway, 1988; Harding, 1991). For their inventors, metaphors often seem apt and sometimes so self-evident that their metaphoricity is not noticed. However, they may be apt only for certain groups and in certain cultural contexts. Feminist researchers especially have argued that it is necessary to trace possible social and material effects of metaphors (e.g., Cowan and Rault, 2022; Haraway, 1991; Suchman, 2008). Many of them aim at reconfiguring “agencies at the human-machine interface” (Suchman, 2008, p. 150). We consider this to be an important future direction, but one that goes far beyond the current goal.

While our findings suggest that it is important to choose explaining metaphors carefully and analyze them for their potentially undesirable implications, it is important to acknowledge limitations that may impact the interpretation or generalizability of our results. Directly eliciting metaphors in the context of a survey is a standard method in metaphor research (Low, 2015). However, this is an artificial situation, and participants may have used metaphors and sentence structures that diverge from those that they would have chosen in a more everyday discourse. From the perspective of *Deliberate Metaphor Theory* (Steen, 2011, 2023), our elicited data likely reflect deliberate metaphors, whereas everyday discourse often relies on non-deliberate metaphors. Differences between elicited and everyday metaphor usage could result in an incomplete picture of how explaining is conceptualized in a real-world context. Future Work should therefore distinguish between spontaneously occurring metaphors and deliberately used ones (Reijnierse et al., 2018).

This research should also be supported by the additional use of corpora (Semino, 2008). For example, scientific texts, newspaper articles or educational books could be analyzed to ascertain whether the metaphorical patterns are consistent or if genre-specific contextual factors have any influence on the explaining metaphors. One study from our group strongly indicates that the same frames with the same low agency dominate scientific texts on explaining/XAI (Scharlau and Rohlfing, 2025).

It should further be noted that explaining metaphors could have only a small or even no framing effect on thinking about explaining. There are two meta-analyses that have compared the effects between non-metaphorical and metaphorical utterances. The results are fairly similar – the effect sizes are small and reliable (*r* = 0.07; Sopory and Dillard, 2002; *r* = 0.09; Van Stee, 2018). Flusberg et al. (2024) also point out that multiple factors, cognitive, social and pragmatic in nature, influence the metaphorical framing effect. Nevertheless, we would argue that because the metaphors we examined are very commonplace and frequent and because implications regarding agency are very similar, they may have a relevant influence on concepts of explaining, expectations of explaining, and on the actual explaining behavior.

Based on *CMT* (Lakoff and Johnson, 1980, 2003) and empirical studies on metaphorical framing (Flusberg et al., 2024; Thibodeau et al., 2017, 2019) it is common and reasonable to assume that explaining metaphors influence thoughts and attitudes about explaining. We have identified a lack of collaboration, constructiveness and bidirectionality in the dominant metaphors of explaining. It should now be investigated whether these metaphors actually lead to less co-constructive views of behaviors in explaining than alternative metaphors that contain all these elements. In the event that different metaphors of explaining do affect the perception of the explaining process differently, the choice of metaphors in explaining contexts, whether in XAI, education, or other domains, should be reconsidered to encourage a more co-constructive interaction.

In addition, future research could investigate whether the effects of agentive framing, as reported by Fausey and Boroditsky (2010), extend to the explanation process. Specifically, it should be analyzed whether the agency of the addressee is valued less if the addressee is not explicitly mentioned in the context of an explanation. If metaphors of explanations predominantly focus on the actions of the explainer, then the role and the agency of the addressee may be backgrounded and therefore reinforce a unidirectional transfer of knowledge.

The similarity of metaphorical patterns observed in both German and English suggests that these metaphors might be deeply embedded in the cognitive and cultural frameworks of explaining. However, this study is limited by its focus on only two closely related languages of western culture. Building on Grady’s proposal (Grady, 1997) of near-universal conceptual metaphors that emerge from bodily experiences, it is plausible that transfer, modification, perception and movement metaphors for explaining occur in other languages. Nevertheless, it seems important to investigate whether these patterns extend to diverse linguistic and cultural backgrounds. In the context of XAI, it is crucial to consider how these culturally embedded metaphors influence user expectations and attitudes toward AI generated explanations.

Finally, one can note that there are ways to ameliorate problematic implications or at least draw attention to them. One of them is the metaphor extension strategy (Landau et al., 2017). This strategy retains the metaphor but adds statements that soften its problematic aspects or make alternative descriptions more prominent. One prominent example is the fight metaphor for cancer that cancer patients often reject because it implies that they have not fought enough if the cancer cannot be stopped. An extension here would be to say that it is a fight with unequal means (Wackers and Plug, 2022). This could be transferred to explaining metaphors, especially those that are so common that they cannot be easily avoided. Explaining can, for example, be still metaphorically described as a process of *giving*, but it could be emphasized that the addressee is actively *taking* the explanandum and may *return* it if it does not match their understanding. Similarly, explaining could be described as the process of *breaking* something *down*, but the process should be described as a collaborative effort. As mentioned above, the domain modification seems to be the most appropriate domain within our data set. The most agentive metaphors within this domain are *building* and *creating*, though the metaphors only emphasize the constructive aspect. The reciprocity and the collaboration would have to be added through extension.

These metaphor extensions and new metaphors which might directly support a co-constructive understanding (think of improvising a piece of music together) may be used in explaining XAI, but also in the future construction of AI systems. This could include developing systems that involve a bidirectional, collaborative, constructive and human-centered conversation, rather than a mere transfer of information. Ultimately, rethinking the metaphors we use for explaining may foster a better communication in both human and AI driven contexts.

## Data Availability

The datasets presented in this study can be found in online repositories. The names of the repository/repositories and accession number(s) can be found at: Open Science Foundation, https://doi.org/10.17605/OSF.IO/Y6SMX.

## Footnotes

^1^For differentiation purposes, conceptual structures are written in small capitals and linguistic metaphors in italics.

^2^ https://doi.org/10.17605/OSF.IO/Y6SMX
