# Supplementary material for: Is explaining more like showing or more like building?—Agency in metaphors of explaining
Source: Front Psychol. 2025 Oct 15;16:1628706. doi: 10.3389/fpsyg.2025.1628706 (PMC12568583; doi:10.3389/fpsyg.2025.1628706)
Supplement: Supplementary file 1 [file Table_1.docx]

| Kodierhandbuch Erklären | | | |
| --- | --- | --- | --- |
| Das Kodierhandbuch stellt einen Leitfaden für die Kodierung von Erklärmetaphern dar. Es enthält die metaphorischen Konzepte, die den Kategorien entsprechen, die in Maxqda kodiert wurden. Die metaphorischen Konzepte sind in alphabetischer Reihenfolge als untergeordnete Kategorien eines metaphorischen Systems aufgeführt. Zu jedem metaphorischen Konzept sind entsprechende Beispiele aus unserem Datensatz angegeben. Die rechte Spalte enthält Hinweise, um mögliche Missverständnisse bei der Kodierung zu vermeiden. | | | |
| Domänen | Metaphorische Konzepte | Definition | Kommentar |
| Transfer | **1. Erklären ist GEBEN**  “Erklären ist eine Information über eine Sache zu *geben*” (17:167f.)  “Wenn man etwas erklärt, *bringt* man Wissen und Information in eine Situation” (248:2981f.)  “Erklären heißt, Wissen *weiterzugeben*” (11:103)  “Informationen [...] *weiterzureichen* (14:151)  “Informations*übergabe*” (12:112) | Jmdm. zum Eigentümer von etw. machen, jmdm. etw. reichen, jmdm., einer Sache etw. zukommen lassen  🡪Bringen  (Etw. (in die Hand) nehmen und an einen Ort tragen, jmdm. Übergeben)  🡪Weitergeben  (Etw. Erhaltenes, Geschenktes einem anderen geben)  🡪Weiterreichen  (Jmdm. eine Sache übergeben, reichen, aushändigen)  🡪 Übergeben  (etw. dem zuständigen Empfänger geben, aushändigen) |  |
|  | **2. Erklären ist LIEFERN**  “[Beim Erklären] sollte man [...] Beispiele *liefern*” (1:3f.) | (Ware) dem Käufer zustellen, an einen bestimmten Ort senden, (postalische Sendungen) dem Empfänger zustellen, überbringen, (etw., das zur Grundversorgung gehört) für Kunden bereitstellen |  |
|  | **3. Erklären ist TRANSFERIEREN**  “Erklären ist wie ein Wissens*transfer*” (208:2485)  “Der erklärende Part [...] möchte dieses Wissen *übermitteln*” (12:115) | [allgemeiner, bildungssprachlich] Übermittlung, Weitergabe (Duden)  🡪 Übermitteln  (Jemandem eine Information senden, zukommen lassen oder als Mittel überbringen) |  |
|  | **4. Erklären ist ÜBERTRAGEN**  “Erklären ist wie eine *Übertragung* von Gedanken” (93:1088) | Über eine Datenleitung, das Internet versenden; über eine Datenleitung, das Internet von einem Ort an einen anderen übermitteln |  |
|  | **5. Erklären ist VERMITTELN**  “Fundamental geht es immer um das *Vermitteln* von neuem Wissen” (42:477)  “Erklären ist wie jemand etwas verständlich *rüberbringen* kann” (40:439) | Jmdm. zu etw. verhelfen, dafür sorgen, dass jmd. etw. erhält  🡪 Rüberbringen  (Vermitteln, weitergeben bzw. (inhaltlich) zugänglich, bewusst, verständlich machen) |  |
| Modification | **6. Erklären ist ANSCHALTEN**  “Erklären ist wie wenn man das Licht *anschaltet*” (170:2033) | (Durch Betätigen eines Schalters, Hebels o. Ä.) in Betrieb setzen, anstellen, einschalten |  |
|  | **7. Erklären ist BAUEN**  “Erklären ist wie eine Brücke *bauen*” (298:3577)  “Erklären ist wie das *Zusammenbauen* von Bausteinen” (41:451) | Etw. durch plangemäßes, festes Zusammenfügen einzelner Teile zu einem bestimmten Zweck für die Dauer errichten, zusammensetzen, etw. schaffen; gestalten  🡪 Zusammenbauen  (Etw. aus einzelnen Teilen bauen, zusammensetzen) |  |
|  | **8. Erklären ist ENTFALTEN**  “Erklären kann man sich wie das *Entfalten* einer Karte [vorstellen]” (118:1393) | Etw. auseinanderfalten, auseinander- breiten |  |
|  | **9. Erklären ist ENTFERNEN**  “Manchmal muss auch etwas, was bereits in diesem Eimer liegt wieder *entfernt* werden” (65:756f.)  “Ziel des Erklärenden sollte es sein alle Unstimmigkeiten bei der Person *auszuräumen*” (27:295f.) | Bewirken, dass etw., jmd. nicht mehr da ist; etw., jmdn. beseitigen  🡪 Ausräumen  (Etw. völlig aus etw. herausräumen; etw. aus dem Wege räumen; beseitigen) |  |
|  | **10. Erklären ist FÜLLEN**  “Erklären ist wie das *Befüllen* eines [...] Eimers” (65:753) | Etw. mit etw.--> vollmachen; etw. in etw. hineintun, hineingießen, hineinschöpfen |  |
|  | **11. Erklären ist GREIFBAR MACHEN**  “Erklären ist [...] der Versuch etwas *begreiflich* zu *machen*” (211:2526f.) | Etw. für jmdn. verfügbar machen | Diese Metapher ist oft unauffällig. |
|  | **12. Erklären ist KLÄREN**  “[Der Erklärende] versucht, bestimmte Informationen zu *klären*” (193:2303ff.)  “Eine Erklärung dient dazu, dem Gegenüber das eigene Wissen *klar zu machen*” (31:337f.)  “Erklären *bringt Klarheit*” (59:689)  “Es ist ein komplexer Prozess, der [...] Klarheit schafft” (71:821f.)  “Erklären ist wie ausdrücken. Etwas soll *verdeutlicht* werden” (156:1864) | Etw., besonders Flüssigkeit oder Luft, von trübenden Bestandteilen frei machen; von trübenden Bestandteilen frei werden  🡪 Klar machen  🡪 Klarheit bringen  🡪 Klarheit schaffen  🡪 Verdeutlichen  (Etw. deutlich, deutlicher, klar, klarer machen) |  |
|  | **13. Erklären ist HINZUFÜGEN**  “Erklären ist wie ein Bild von etwas zu zeichen. Man beginnt mit einem ersten Strich, *fügt* dann immer weitere *hinzu* [...]” (141:1660f.)  “Erklärungen [...] können das Zeigen von Abläufen auch unterstützen und *ergänzen*” (173:2074f.) | Etw. zu etw. legen, dazufügen  🡪 Ergänzen  (etw. hinzufügen) |  |
|  | **14. Erklären ist LÖSEN**  “Ein Erklärer hat die Aufgabe, den Empfänger durch das *Lösen* eines Rätsels zu führen” (183:2188f.) | Etw. entwirren, auflösen, [bildlich] enträtseln |  |
|  | **15. Erklären ist MALEN**  “Erklären ist wie *malen*” (154:1837)  “Erklären ist wie das *Zeichnen* eines Bildes” (114:1335) | ein Bild schaffen; etw., jmdn. in Form eines Bildes darstellen  🡪 Zeichnen  (Eine (technische, künstlerische) Zeichnung von etw., jmdm. schaffen, anfertigen |  |
|  | **16. Erklären ist ÖFFNEN**  “Erklären ist wie das *Öffnen* einer Tür” (135:1586) | Etw. Zugeschlossenes aufschließen; etw. Geschlossenes, Zugemachtes aufmachen |  |
|  | **17. Erklären ist ORDNEN**  “Erklären ist wie [...] ein Blatt mit vielen verdrehten Wörtern richtig zu *ordnen*” (123:1443f.)  “Erklären ist ein Puzzle an Informationen *anzuordnen*” (306:3687)  “Erklären ist wie die Vermittlung von [...] Wissensgrundsätzen und deren *Einordnung*” (201:2404f.)  “Erklären [ist] sprachlich *Ordnung schaffen*” (14:143f.) | Etw. an einem bereits vorgesehenen Platz in einer bestimmten Reihenfolge unterbringen, Gegenstände, Ereignisse, Begriffe erfassen und systematisch zusammenfügen  🡪 Anordnen  (etw. nach bestimmten Gesichtspunkten ordnen, einrichten, aufstellen)  🡪 Einordnen  (An einen passenden Platz in einem vorstrukturieren System einfügen; in ein vorstrukturiertes System einsortieren)  🡪 Ordnung schaffen | Achtung:  „In Teile auf- teilen“ ist eine andere Metapher |
|  | **18. Erklären ist PLATZIEREN**  “Der Erklärende muss sicherstellen, dass jeder Teil an der richtigen Stelle [...] *platziert* wird” (183:2192f.) | Jmdn., etw. an einen bestimmten Platz setzen, stellen, legen, an einem bestimmten Platz unterbringen |  |
|  | **19. Erklären ist SCHAFFEN**  “Erklären ist also ein geordnetes Beschreiben [...], um [...] ein klares Bild zu *schaffen*” (102:1197f.)  “[Erklären ist] [...] etwas künstlerisches zu *erschaffen*” (66:770ff.) | Etw. (schöpferisch, gestaltend) hervorbringen, entstehen lassen  🡪 Erschaffen  (Etw., jmdn. durch eigene schöpferische Kraft entstehen lassen, schaffen) |  |
|  | **20. Erklären ist VERBREITEN**  “Erklären ist wie ein Prozess [...] um Wissen zu *verbreiten*” (153:1825) | Etw. allgemein, vielen bekannt machen; etw. dehnt sich über ein größeres Gebiet aus |  |
|  | **21. Erklären ist VERKNÜPFEN**  “Es geht also darum bestehendes Wissen mit neuen Dingen zu *verknüpfen* “ (42:471f.) | Von Fäden, Schnüren, Seilen, Bändern: durch Knoten, Schlaufen o. Ä. verbinden, festmachen, verknoten; (zwei oder mehrere Dinge) inhaltlich verbinden, in einen Zusammenhang bringen |  |
|  | **22. Erklären ist ZERLEGEN**  “Erklären ist wie das *Zerlegen* eines [...] komplexen Sachverhaltes” (189:2256)  “Man [...] *nimmt* die einzelnen Komponenten des Gesagten *auseinander*” (213:2545f.) | Die Bestandteile von etw. Zusammengesetztem einzeln aus ihrem Gefüge lösen  🡪Auseinandernehmen  (In einzelne Bestandteile, kleinere Einheiten zerlegen) |  |
|  | **23. Erklären ist ZUSAMMENSETZEN**  “Erklären ist wie ein Puzzle *zusammensetzen*” (266:3192)  “Erklären ist wie das *Zusammenfügen* von einzelnen Puzzlestücken” (102:1191)  “Erklären ist für mich wie das *Zusammenstellen* eines Puzzles” (155:1848) | Etw. aus Einzelteilen zu einem Ganzen verbinden  🡪 Zusammenfügen  (Etw. zusammensetzen, zu einem Ganzen verbinden)  🡪 Zusammenstellen  (Etw. aus mehreren ausgewählten Teilen zu einem Ganzen zusammensetzen, gestalten) |  |
| Perception | **24. Erklären ist BELEUCHTEN**  “Erklären ist wie das *Beleuchten* von dunklen Stellen” (164:1959)  “Erklären ist wie wenn man das Licht *anschaltet*” (170:2033)  “Etwas mit Informationen *ausleuchten*” (15:147f.)  “Erklären ist wie ein strahlendes Feuer in einer finsteren Nacht, das *Licht auf das Unbekannte wirft* und den Weg des Wissens *erhellt*” (194:2314f.)  “Erklären ist wie *Licht ins Dunkle zu bringen*” (63:731) | Auf etw., jmdn. Licht werfen; etw. erleuchten, hell machen  🡪 Ausleuchten  (Etw. bis in den letzten Winkel beleuchten, einen Raum ganz mit Licht erfüllen)  --> Erhellen  (Etw. hell machen, erleuchten)  🡪 Licht ins Dunkle bringen  🡪 Licht auf etwas werfen |  |
|  | **25. Erklären ist SUCHEN**  Erklären ist wie wenn man [...] die richtigen Worte zusammen *suchen* muss (73:843f.) | Sich bemühen, jmdn. oder etw. Bestimmtes, was man haben möchte, braucht, verloren hat, zu finden |  |
|  | **26. Erklären ist ZEIGEN**  “Erklären ist wie *Zeigen*” (50:574)  Unter "erklären" verstehe ich nun, dass ich die Hintergründe zu diesem Prozess *aufzeige* (119:1505-1506)  “Die Erklärung muss [..] in geeigneter Weise *präsentiert* werden” (225:2703f.) | Den Finger, den Arm auf die Stelle, den Ort, an dem sich etw. befindet, richten, um darauf aufmerksam zu machen  🡪 Aufzeigen  (Etw. im Einzelnen nachweisen, vor Augen führen)  🡪 Präsentieren  (Zeigen, vorführen, vorstellen, bekannt machen) (Duden) |  |
| Motion | **27. Erklären ist BEGLEITEN**  “Man kann ihnen den Schlüssel geben und sie in diese neue Wissenswelt *begleiten*” (77:892f.) | Mit jmdm. mitgehen, jmdm. Gesellschaft leisten; jmdn. beratend unterstützen |  |
|  | **28. Erklären ist GEHEN**  “Erklären ist wie jemanden an die Hand nehmen und den Weg [...] mit ihr [zu] *gehen*” (163:1948f.) | Sich zu Fuß fortbewegen; sich irgendwohin begeben |  |
|  | **29. Erklären ist FÜHREN**  “Erklären ist wie jemanden auf den Pfad zu neuem Wissen zu *führen*” (93:1083f.) | Jmdn. leiten, lenken |  |
|  | **30. Erklären ist MITNEHMEN**  “Der Erklärende *nimmt* den Erklärungsempfänger *mit* auf eine Reise” (235:2819f.) | Etw. oder jmdn. mit sich fortnehmen |  |
