# Supplementary material for: Is explaining more like showing or more like building?—Agency in metaphors of explaining
Source: Front Psychol. 2025 Oct 15;16:1628706. doi: 10.3389/fpsyg.2025.1628706 (PMC12568583; doi:10.3389/fpsyg.2025.1628706)
Supplement: Supplementary file 2 [file Table_2.docx]

| Coding Manual Explaining | | | |
| --- | --- | --- | --- |
| The coding manual provides guidance on how to code metaphors of explaining. It contains the metaphorical concepts that correspond to the categories that have been coded in MAXQDA. The metaphorical concepts are listed in alphabetical order as subordinate categories of a metaphorical system. The definitions are mainly taken from the *Macmillan English Dictionary.* Where this was not sufficient, the *Oxford Advanced Learner's Dictionary* was used, which is indicated by the abbreviation OALD*.* For each metaphorical concept there are corresponding examples from our data set. The right-hand column contains notes to avoid possible misunderstandings during coding. | | | |
| Domains | Frames | Definition | Comment |
| Transfer | **1. Explaining is CONVEYING**  ‘an explainer uses different methods [...] to *convey* information and ideas’ (30:326) | To give official information or a formal message to someone | Do not code: Transferring  (To copy information or images from one place or object to another) |
|  | **2. Explaining is DELIVERING**  ‘explanations *deliver* more than just simple factual answers’ (236:2745) | To take something, especially goods or letters, to a place |  |
|  | **3. Explaining is GIVING**  ‘you are *giving* other examples’ (2:13)  ‘explaining is *elaborating* and providing more detail and information’ (29:320)  ‘you are actively trying to *impart* knowledge’ (81:914f.)  ‘to *pass on* what you know to someone’ (173:2016)  ‘an explanation is [...] *providing* layers of information’ (28:305)  ‘it's about *sharing* new knowledge and ideas’ (107:1223f..) | To put something in someone’s hand, or to pass something to someone  🡪 Elaborating  (To give more details or information about something)  🡪Impart  (To give something such as information, knowledge or beliefs to someone)  🡪Passing On  (To give someone something that someone else has given you)  🡪Providing  (To give someone something that they want or need)  🡪Sharing  (To give a part of something to someone else) | Do not code: Bringing |
| Modification | **4. Explaining is ADDING**  ‘explaining is like *adding* branches to a tree’ (210:2432)  ‘you are *supplementing* his knowledge’ (4:46f.) | To put something with another thing or group of things  🡪Supplementing  (To add something extra in order to improve something or make it bigger) | Do not code: To state or explain something |
|  | **5. Explaining is ADJUSTING**  ‘a good explainer *adjusts* their approach’ (175:2036f.)  ‘such as analogies [...] that an explainer might use to help *shape* the information into a clear and understandable form’ (30:328f.)  ‘a good explainer will *tailor*  their approach to the person they are trying to reach’ (87:992f.) | To change something slightly in order to make it better, more accurate, or more effective  🡪Shaping  (To form something into a particular shape)  🡪Tailoring  (To make or change something especially for a particular person or purpose) |  |
|  | **6. Explaining is BREAKING DOWN**  ‘it involves *breaking down* complex ideas [...] into simpler, more manageable parts’ (39:436f.)  ‘explaining is like *deconstructing* a concept’ (7:69)  ‘explaining is like *taking* something *apart’* (170:1973) | To divide something such as a total amount into separate parts  🡪Deconstructing  (To separate something into the parts from which it is made up and put them together again in a different way) (OALD)  🡪Take apart  (Separate an object into pieces) |  |
|  | **7. Explaining is BUILDING**  ‘explaining can be like *building* a bridge’ (165:1916)  ‘explaining is like *constructing* a bridge’ (225:2622) | To make a building or larger structure by putting its parts together  🡪Constructing  (To build something large or complicated, such as a bridge or a road) |  |
|  | **8. Explaining is CONNECTING**  ‘explaining is a bridge that *connects* the unknown and the known’ (57:636) | To join two things together |  |
|  | **9. Explaining is CREATING**  ‘explaining is attempting to [...] *create* a picture or image of an object’ (40:445)  ‘as a sculptor uses different tools [...] to shape and *mould* the marble [...], an explainer uses different methods and approaches to convey information’ (30:325f.) | To make something new or original that did not exist before  🡪Moulding  (To give something a particular shape or form) |  |
|  | **10. Explaining is MAKING**  ‘explaining is like *making* sense of a situation’ (35:382) | To create or produce something by working |  |
|  | **11. Explaining is OPENING**  ‘explaining is like *opening* up a suitcase’ (4:36) | To move a door or window into a position that allows people or things to pass through; to separate the edges of something, or to take off its cover so that you can see or remove what is inside |  |
|  | **12. Explaining is PAINTING**  ‘when [...] explaining something you *paint* a vivid picture in someones head’ (80:902)  ‘explaining is like *drawing* a picture for someone’ (80:901) | To create a picture of something using paints  🡪Drawing  (To create a picture by making lines with a pen or pencil) |  |
|  | **13. Explaining is PUTTING TOGETHER**  ‘it’s like *putting* pieces of a puzzle *together’* (70:789)  ‘to explain something means to [...] *combine* [individual pieces of something] into a whole’ (208:2419f.)  ‘[...] take small pieces of a whole and *fit* them *together’* (184:2134f.)  *‘piecing* a topic *together’* (184:2140) | To produce or organize something using many different things; to make something by joining all its parts  🡪Combining  (If you combine things, or if they combine, you use, do, or put them together)  🡪Fitting Together  (If you fit things together, you join them in the right way to make something)  🡪Piece Together  (To make something by combining separate bits) |  |
|  | **14. Explaining is REMOVING**  ‘explaining is like *removing* clouds of ignorance’ (236:2742)  ‘explaining is like *peeling* an onion’ (77:869)  ‘an explanation is *taking* little bits *out* of the lake’ (28:304f.)  ‘explaining is *taking away* the unnecessary information’ (28:310)  ‘explaining is like *unpacking* something’ (54:603) | To take something or someone away from a place  🡪Peeling  (To remove skin from a fruit or vegetable; to remove something from the surface of something else, especially by taking one end or side and pulling it up)  🡪Taking out  (To remove something)  🡪Taking away  (To remove something)  🡪Unpacking  (To take things out of a suitcase or other container that you were carrying them in) |  |
|  | **15. Explaining is SOLVING**  ‘explaining is [...] *solving* things and problems that arise’ (35:389f.)  ‘to explain what was actually said to *resolve* the issue’ (131:1499f.) | To find a solution to something that is causing difficulties; to find the answer to a question in a puzzle, crossword etc.  🡪Resolving  (To solve a problem or to find a satisfactory way of dealing with a disagreement) | Pay attention to context; it’s often a consequence |
|  | **16. Explaining is TURNING ON**  ‘explaining is like *turning* the lights *on’* (149:1726) | To make a piece of equipment start working by pressing a button or moving a switch |  |
| Perception | **17. Explaining is CLARIFYING**  ‘explaining is to *clarify* a concept’ (77:868)  ‘you have to *clear* it *up* for them’ (109:1341f.)  ‘you would need to *make* all the facts *clear’* (27:299) | To make something clearer or easier to understand (OALD)  🡪Clearing up  (To make something clean and neat) (OALD)  🡪Making Clear |  |
|  | **18. Explaining is ILLUMINATING**  ‘explaining is [...] *illuminating* the way (39:440f.)  ‘explaining also helps to *shed* some *light* to a confusing idea’ (149:1730f.)  ‘explaining is like *shining a light* on a dark path’ (39:440f.) | To make a bright with light, or to shine a light on something  🡪Shedding a light  (To send light over something; to let light fall somewhere) (OALD) | Do not code: Highlighting |
|  | **19. Explaining is SEEING**  ‘explaining can also be described as *seeing’* (196:2276) | To notice someone or something using your eyes | Do not code: Looking |
|  | **20. Explaining is SHOWING**  ‘you can *show* the other person what you mean’ (2:17f.)  ‘explaining is like [...] *pointing out’* (188:2184f.)  ‘explaining [...] is a clear method of *portraying* [...]’ (204:2365f.)  ‘explaining is like *presenting* information’ (212:2459)  ‘explaining can also just be *revealing* information or facts’ (212:2465) | To let somebody see something  🡪Pointing out  (To show something by holding out your finger or a long thin object)  🡪Portraying  (To show or describe someone or something in a particular way)  🡪Presenting  (To show someone or something in a particular way so that people have a particular opinion about them/that)  🡪Revealing  (To show something that was covered or hidden) | Do not code: To give someone an instruction or an explanation |
| Motion | **21. Explaining is GUIDING**  ‘when you explain something, you are *guiding* someone through a new idea’ (97:1105f.) | To act as guide to; to go with or before for the purpose of leading the way (OALD) | Do not code: Explaining is like being a tour guide |
|  | **22. Explaining is GOING**  ‘it means to inform someone and *go* beyond just a word’ (4:37)  *‘going* further and further into detail’ (25:266) | To move or travel to a place that is away from where you are now | Do not code when it is an auxiliary verb |
|  | **23. Explaining is TAKING**  ‘when you explain something, you *take* someone on a journey’ (112:1274) | To move something or someone from one place to another |  |
